# A Wide QRS Tachycardia with Three Distinct Left Bundle Branch Block Morphologies in a Patient with Sinus Rhythm with Left Bundle Branch Block: What Is the Mechanism?

**DOI:** 10.19102/icrm.2021.121007

**Published:** 2021-10-15

**Authors:** Bulent Deveci, Meryem Kara, Ahmet Korkmaz, Ozcan Ozeke, Serkan Cay, Firat Ozcan, Serkan Topaloglu, Dursun Aras

**Affiliations:** ^1^Department of Cardiology, University of Health Sciences, Ankara City Hospital, Ankara, Turkey

**Keywords:** Idiopathic ventricular tachycardia, left bundle branch morphology, left bundle branch block tachycardia, left and right cusp commissure, preferential conduction

## Abstract

The differential diagnosis for a wide complex tachycardia includes all causes of supraventricular tachycardia (SVT) with bundle branch block or all causes of SVT with antegrade pre-excitation by bystander involvement of any accessory pathways, myocardial or bundle brunch ventricular tachycardia, and antidromic (atriofascicular or nodofascicular/nodoventricular) and other pre-excited reciprocating tachycardias. We present a case of wide complex QRS tachycardia with a left bundle branch block QRS morphology.

## Case presentation

**Figure 1:**
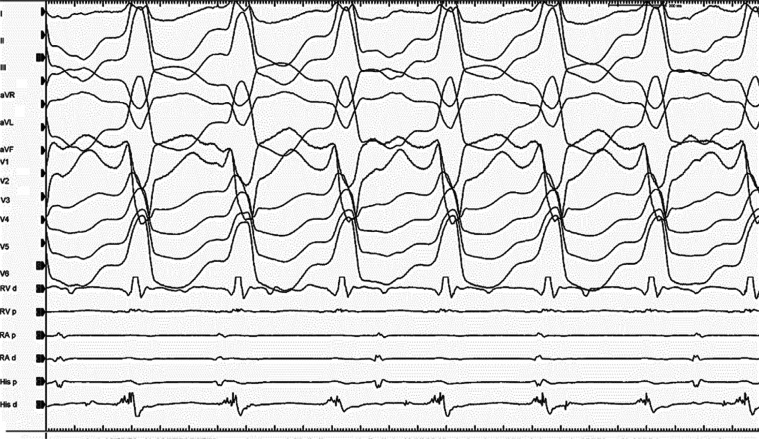
Left bundle branch block morphology tachycardia is seen (VT1) with dissociated atrial signals that are followed by His signals. d: distal; p: proximal; RA: right atrium; RV: right ventricle.

**Figure 2:**
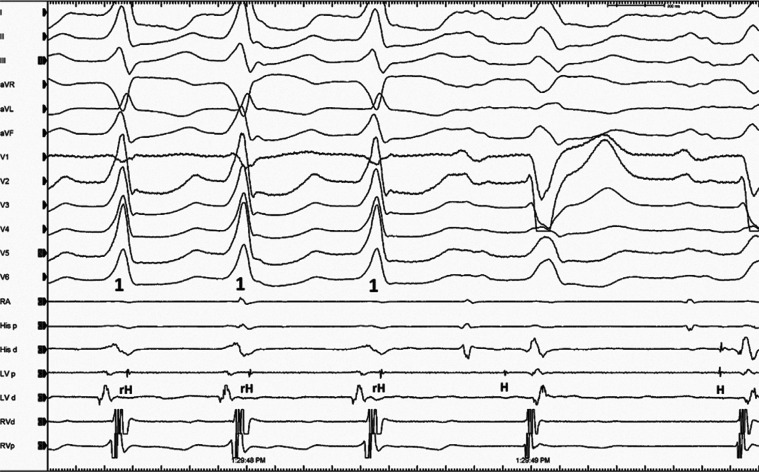
The termination of second wide QRS tachycardia (VT2) is seen. d: distal; LV: left ventricle; p: proximal; RA: right atrium; RV: right ventricle.

A 45-year-old woman with recurrent wide complex QRS tachycardia was admitted to our hospital for an electrophysiological study. Echocardiography showed no structural abnormality; however, cardiac magnetic resonance imaging reported a patchy distribution of cardiac late gadolinium enhancement in the periaortic region. She had a clinically documented episode of wide complex QRS tachycardia with a left bundle branch block QRS morphology. During the electrophysiological study, the fragmented signals were seen on the His electrogram during tachycardia **(Figure 1)**. A second wide complex QRS tachycardia with a QS pattern with a descending limb notching in lead V1 developed soon after **(Figure 2)**. Later, a transient third wide complex QRS tachycardia was also observed. What is the mechanism of these tachycardias?

## Discussion

The differential diagnosis for a wide complex QRS tachycardia includes all causes of supraventricular tachycardias with a bundle branch block, or all causes of supraventricular tachycardias with an antegrade preexcitation by bystander involvement of any accessory pathways, myocardial or bundle branch ventricular tachycardias (VTs), and antidromic (atriofascicular or nodofascicular/nodoventricular) and other pre-excited reciprocating tachycardias.^1,2^ Isolated periaortic substrates should also be suspected in patients with nonischemic cardiomyopathy with minimally depressed left ventricular ejection fraction without an overt scar or wall motion abnormalities presenting with VT with an inferior axis.^3^ Therefore, distinguishing between an antegrade His and a retrograde His is a critical step in the interpretation of wide complex QRS tachycardia cases.^4^ Antegrade activation suggests that the mechanism of the wide complex QRS tachycardia is a supraventricular tachycardia with aberrancy, whereas retrograde activation is consistent with either VT or antidromic tachycardias, including atriofascicular or nodofascicular/nodoventricular reentrant tachycardias.^4^ The dissociation of antegrade His signals from the ongoing tachycardia excluded the nodofascicular/nodoventricular or bundle branch participation in the current case **(Figure 1)**. Furthermore, **Figure 1** demonstrated a continuation of the tachycardia despite atrioventricular dissociation. This provided proof that the atrium was not necessary to sustain the tachycardia and ruled out the participation of accessory pathways.^5^ The principal unique maneuver in the differential diagnosis of the wide complex QRS tachycardia is the placement of sensed premature atrial extrasystole during the tachycardia, the pacing of the atrium faster than the tachycardia to entrain the tachycardia, and then the analysis of the return beats after cessation of pacing.^6^ In the current case, both early and late atrial extrasystoles did not affect the morphology and cycle length of the ventricular electrograms during tachycardias.^7,8^ Comparison of the QRS complexes between the tachycardia and those in sinus rhythm is also helpful as a QRS complex that is narrower during wide complex QRS tachycardia than during sinus rhythm establishes the diagnosis of VT **(Figure 2)**.^9–11^

**Figure 3:**
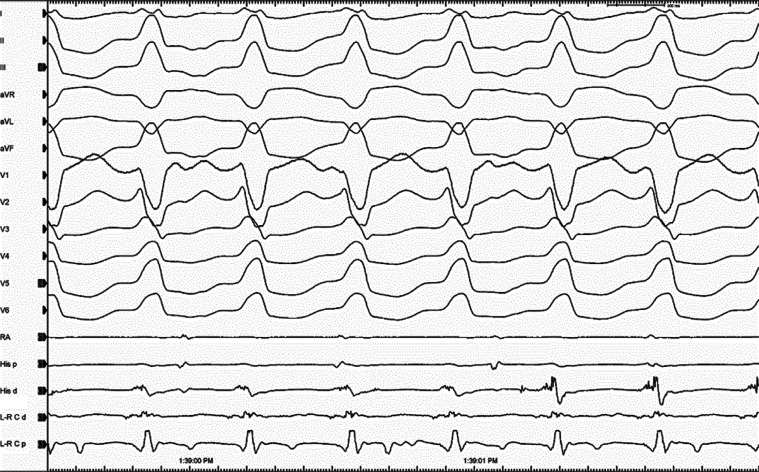
The electrograms during the first tachycardia (VT1) are seen. Compare also the earliest ventricular activation time between the L-R C d and His d channels. d: distal; L-R C: left and right cusp commissures; p: proximal; RA: right atrium; RV: right ventricle.

**Figure 4:**
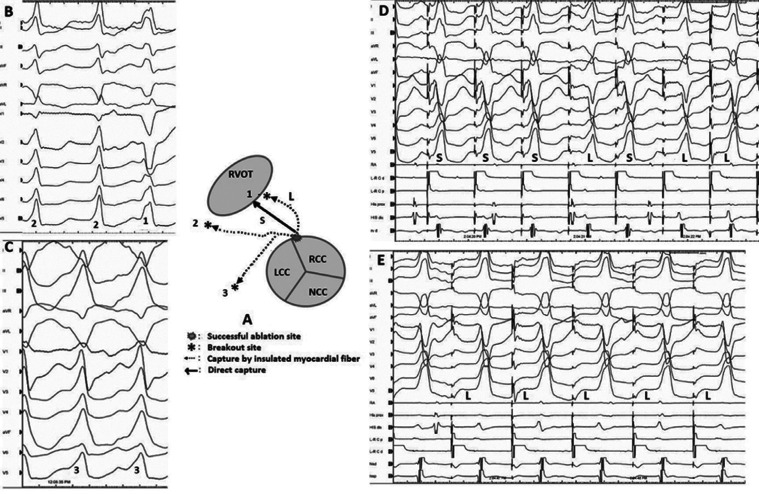
**A:** The concept of preferential conduction routes is seen. The (1) shows first ventricular tachycardia morphology (VT1 in **B**), (2) shows the second VT morphology (VT2 **B**), and (3) shows third ventricular tachycardia morphology (VT3 in **C**). Note the short and long stimulus-to-QRS intervals (**D** and **E**) almost suggesting direct capture (S, short S-to QRS) and the capture via an insulated myocardial fiber across the ventricular outflow septum (L, long S-to QRS). L: the long stimulus to QRS interval; S: the short stimulus to QRS interval. RV: right ventricle; RA: right atrium; L-R C: left and right commissures; p: proximal; d: distal.

**Figure 5:**
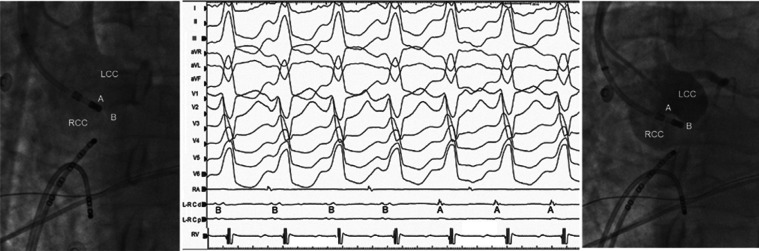
Note the far-field (B points → the initial four electrograms taken below the L-R C) and near-field signals (A points → the last three electrograms taken above the L-R C) at successful ablation point. d: distal; LCC: left coronary cusp; L-R C: left and right commissure; p: proximal; RA: right atrium; RCC: right coronary cusp; RV: right ventricle.

We started mapping from the right ventricular outflow tract due to the delayed QRS transition in the precordial leads **(Figure 1)**. However, the first tachycardia converted to a second tachycardia without the completion of activation mapping. According to the characteristics of a QS pattern with a notch on the descending limb in lead V1 **(Figure 2)**, the second VT was initially considered to be arising from the commissure between the left and right coronary cusps (L-RCC).^12–14^ We continued the mapping from the aortic cusps and detected the earliest ventricular activation with fractionated signals in the L-RCC region **(Figure 2)**. Interestingly, this place was also the earliest ventricular activation point for the first wide complex QRS tachycardia **(Figure 3)**. A third VT **(Figure 4C)** was also seen, but it was infrequent. Pace-mapping from the L-RCC region with high and low voltages^14–17^ showed similar QRS morphologies with all three VTs and short and long stimuli (S) to QRS intervals, with most of them suggesting direct capture (short S to QRS interval), and the capture via an insulated myocardial fiber across the ventricular outflow septum (long S to QRS interval) (**Figures 4D and 4E**, respectively). Therefore, all these three wide complex QRS tachycardias were thought to originate from the L-RCC with three breakout sites **(Figure 4)** compared to the localized reentry confined to this anatomically challenging region,^3,18–20^ suggesting the existence of preferential conduction from the L-RCC commissure to both the right and left outflow tracts. The first radiofrequency lesion below the L-RCC was suppressed transiently (B points in **Figure 5**), but the application from near-field potentials taken above the L-RCC (A points in **Figure 5**) terminated the tachycardia. Burst pacing and isoproterenol failed to elicit any further tachycardia. The other clinical VT was also no longer seen. This was a simple tracing, with multiple findings that instructively show the importance of both preferential conduction and the pacing threshold to define preferential conduction routes in the identification of successful ablation points.
